# The Productivity of Medical Publication on COVID-19 in the First Half of 2020: A Retrospective Analysis of Articles Available in PubMed

**DOI:** 10.7759/cureus.11814

**Published:** 2020-11-30

**Authors:** Bartosz M Nowak, Mikołaj Kamiński

**Affiliations:** 1 Medicine, Poznan University of Medical Sciences, Poznań, POL; 2 Internal Medicine and Diabetology, Independent Public Clinical Hospital I, Szczecin, POL

**Keywords:** pubmed, covid 19, productivity, coronavirus, medline

## Abstract

Background

The control of the coronavirus disease 2019 (COVID-19) pandemic depends on the profound investigation of the virus biology and its consequences. We aimed to analyze the COVID-19 research productivity of authors representing different countries and associations between the number of articles and COVID-19 spread.

Methods

We retrieved all articles on COVID-19 indexed in PubMed between 31 December 2019 and 30 June 2020. We identified the countries of individual authors’ affiliations. We performed the R Spearman rank correlation test between the number of articles with at least one author from a country per one million citizens and Human Development Index (HDI), a number of COVID-19 cases and deaths per one million citizens before 1 July 2020.

Results

Overall, we identified 27,815 articles, including 18,225 original contributions, 2,449 reviews, and 69 meta-analyses on severe acute respiratory syndrome coronavirus 2 (SARS-CoV-2) infection. The highest productivity characterized the authors coming from China (n = 11,519 articles with at least one author), followed by the United States of America (n = 9,666) and Italy (n = 7,261). The number of articles on COVID-19 associated with HDI (Rs = 0.79), the numbers of cases (Rs = 0.47), and deaths (Rs = 0.46) (all p < 0.001).

Conclusions

Early COVID-19 researches were most often authored by researchers from highly developed countries and those affected by the rapid initial spread of SARS-CoV-2.

## Introduction

Coronavirus disease 2019 (COVID-19) pandemic is a novel public health emergency, activating the global healthcare system. The control of the pandemic and treatment of people affected depends on deep understanding of the infection's biology and course. Since the first reports of pneumonia of unknown origin in Wuhan [1], numerous researchers started investigations on the novel etiological agent. Currently, over half a year has passed since the pandemic was declared by the World Health Organization. There is little knowledge on the productivity of medical researches during this period. Herein, we describe the sources of the outpour of publications on COVID-19. The scientific productivity in the early stages of a public health emergency may reflect the health institutions’ adaptability and indicate areas of focus for the future. We hypothesized that the analysis may reveal which countries lead in the investigations on the virus and indicate the main directions of research.

We aimed to analyze articles on COVID-19 available via PubMed in the first half of 2020 in order to (1) compare scientific productivity of authors representing different countries and (2) explore associations between the number of articles and COVID-19 spread.

## Materials and methods

Data collection, manipulation, calculations, and visualization were performed using the R (version 3.6.3) programming language (R Foundation, Vienna, Austria). We collected data on COVID-19 publications using the R PubMed API called "easyPubMed" on 9th of July 2020 [2]. We set the following query conditions: date between the 31st of December 2019 and 30th of June 2020, and search terms: "novel coronavirus," "coronavirus Wuhan," "SARS-CoV-2," "COVID-19." We identified articles with or without abstract, original articles, reviews, meta-analyses, and guidelines.

We ranked countries based on the number of articles with at least one author coming from each country. We confronted the ranking of countries with the highest number of articles on COVID-19 with SCImago ranking of countries with the highest medical researchers' productivity in 2019 [3]. Moreover, we conducted the Spearman rank correlation test between the number of articles in each country and:

(a) the total number of COVID-19 cases per one million citizens before the 1st of July 2020 [4],

(b) the total number of deaths related to COVID-19 per one million citizens before the 1st of July 2020 [5], and

(c) Human Development Index (HDI) for 2018 [6].

We choose the R Spearman correlation rank test because the data we analyzed is presented in interval scale (e.g., HDI is an artificial index, and similarly like BMI, non-parametrical tests should be preferred). Our data also had many outliers, which is other indication of Spearman correlation rank usefulness. We matched journal names with the SCImago journal database that contains information on the journal category [7]. For instance, "The Lancet Infectious Diseases" is categorized as "Infectious Diseases" journal, while "Clinical Microbiology Reviews" as one of the following: "Epidemiology," "Infectious Diseases," "Microbiology (medical)," or "Public Health, Environmental and Occupational Health" journal. We counted the number of reports on COVID-19 in journals from each category and presented categories with the highest number of publications.

## Results

In the first half of 2020, the total number of articles published on PubMed equaled 858,641. Overall, we identified 27,815 articles on COVID-19, which constitutes 3.24% of all publication in this time. Among those we found 18,225 (65.52%) original articles, 2,449 (8.8%) reviews, 69 (0.25%) systematic reviews with meta-analysis, and 171 (0.61%) guidelines. The remaining 6,901 (24.81%) positions were letters to the editors, commentaries, errata, or unclassified. In a per-month analysis we found that in January, two articles (0.007%) on COVID-19 were indexed, 37 (0.13%) in February, 620 (2.23%) in March, 2,514 (9.04%) in April, 5,527 (19.87%) in May, and 18,596 (66.85%) in June. From those articles, we retrieved n = 519 preprint publications (n = 319 from medRxiv and n = 200 from bioRxiv).

We were able to identify 62,051 authors coming from 148 countries. Most authors came from China (n = 11,519), followed by the United States of America (n = 9,666) and Italy (n = 7,261) (Table 1).

**Table 1:** Top 20 countries with the highest number of publications on COVID-19 between 31 December 2019 and 30 June 2020.

Rank	Country	Number of publications with at least one author from the country	SCImago country rank
1	China	11,519	2
2	United States of America	9,666	1
3	Italy	7,261	6
4	United Kingdom	4,362	3
5	France	3,459	9
6	India	2,568	10
7	Spain	1,765	11
8	Canada	1,679	7
9	Germany	1,596	4
10	Iran	1,349	17
11	Australia	1,317	8
12	Singapore	1,307	34
13	Brazil	1,026	14
14	Turkey	924	16
15	Switzerland	796	15
16	Netherlands	753	12
17	Japan	717	5
18	Taiwan	643	23
19	Belgium	529	20
20	Israel	459	26

**Figure 1:**
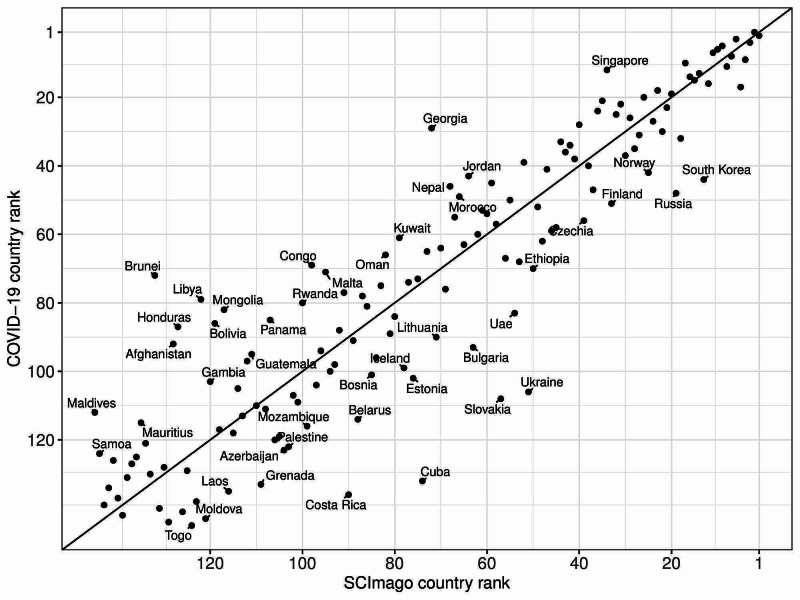
Comparison of SCImago country rank in category medicine and the rank of the countries with the highest number of articles on COVID-19.

In most cases, the country rank based on the number of publications on COVID-19 was similar to the SCImago country rank (Figure 1).

**Figure 2:**
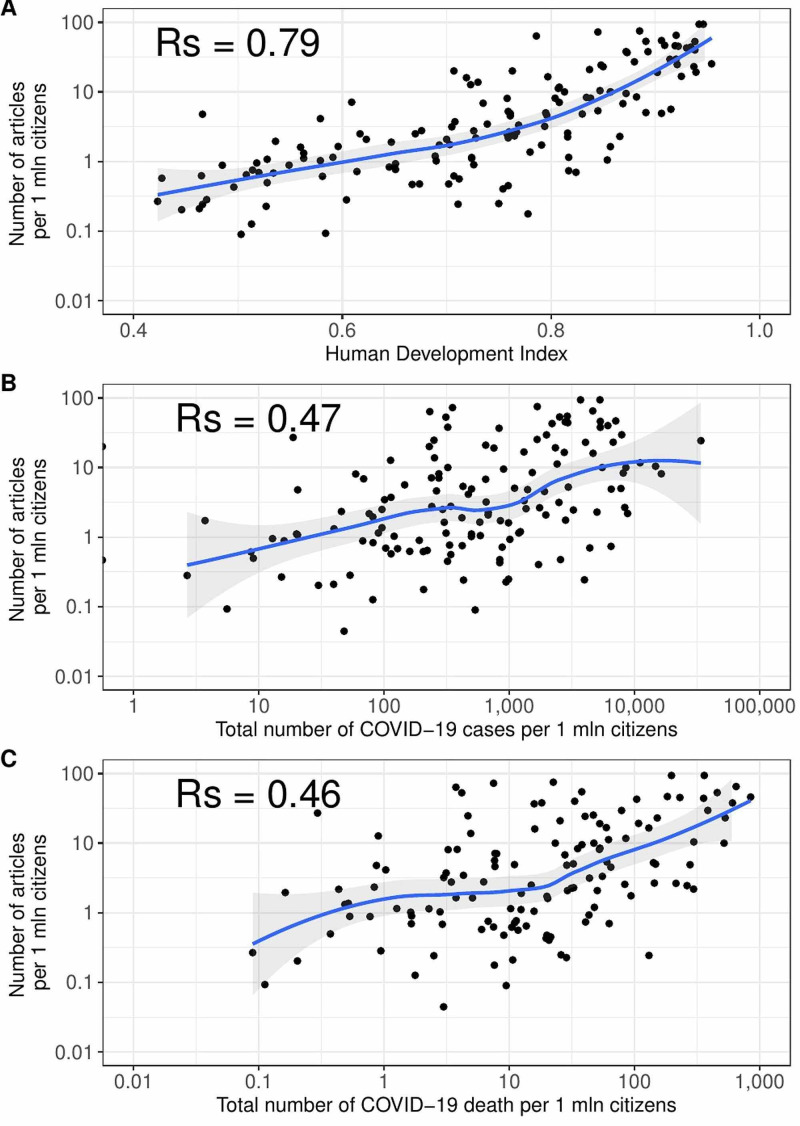
Correlation plots between number of articles one author from a country per at least one million citizens and (A) Human Development Index, (B) total number of COVID-19 cases per one million citizens, and (C) total number of COVID-19 deaths per one million citizens.

The number of articles per one million citizens was positively associated with the Human Development Index, number of cases, and deaths due to COVID-19 per one million inhabitants (all p < 0.001) (Figure 2).

We identified 3,156 unique journal names to which we could match 2,571 titles (80.4%), including 27,296 unique articles that we were able to retrieve. We found that most of the matched journals belonged to category “Medicine” (n = 5,038), followed by “Infectious Diseases” (n = 2,204) and “Cardiology and Cardiovascular Medicine” (n = 1,170) (Table 2).

**Table 2:** Top 20 journal categories with the highest number of articles on COVID-19 till 30 June 2020.

Rank	Categories	Number of articles
1	Medicine	5,038 (21.18%)
2	Infectious Diseases	2,204 (9.26%)
3	Cardiology and Cardiovascular Medicine	1,170 (4.92%)
4	Dermatology	708 (2.98%)
5	Public Health, Environmental and Occupational Health	531 (2.23%)
6	Neurology	525 (2.21%)
7	Gastroenterology	514 (2.16%)
8	Anesthesiology and Pain Medicine	511 (2.15%)
9	Biochemistry, Genetics, and Molecular Biology	488 (2.05%)
10	Critical Care and Intensive Care Medicine	472 (1.98%)
11	Internal Medicine	436 (1.83%)
12	Hematology	434 (1.82%)
13	Pediatrics, Perinatology, and Child Health	433 (1.82%)
14	Surgery	425 (1.79%)
15	Multidisciplinary	415 (1.74%)
16	Immunology	396 (1.66%)
17	Health Policy	347 (1.46%)
18	Radiology, Nuclear Medicine, and Imaging	337 (1.42%)
19	Epidemiology	323 (1.36%)
20	Otorhinolaryngology	299 (1.26%)

## Discussion

In this report, we analyzed COVID-19-related publication productivity based on articles available via PubMed in the first half of 2020.

COVID-19-themed items considerably contributed to overall medical scholarly output in this period. Importantly, most of the papers were original articles-the most essential medical knowledge source. However, one in four positions on COVID-19 were minor papers such as editorials, commentaries, etc. The growth in the number of articles was approximately geometrical. These results have to be taken with caution because preprints were indexed in PubMed with delay resulting from the publication process.

COVID-19 pandemic is a new health phenomenon, which requires detailed investigations. Several factors may enhance COVID-19 publication productivity. First, many journals established free open-access for papers on COVID-19 [7]. Moreover, the most valuable reports may also be rapidly reviewed. Many governmental and private institutions donated funds for research on SARS-CoV-2 and its spread [8-10]. Finally, media coverage of scientific progress related to COVID-19 remains intense [11,12]. In these circumstances, researchers have additional motivation to rapidly publish their results.

Every health crisis, like ongoing coronavirus pandemics, requires an acceleration in generating knowledge. To further hasten the research, more funding is required to benefit researchers working on emerging health problems. Other actions taken by authorities could be increasing the scientists' work time or engaging young researchers, who could increase not only their experience but also relieve more practiced scientists from their current projects. Both of these actions require additional funding as well [13]. We should carefully analyze regular scientific expenditure and additional funding available during COVID-19 pandemics to validate this hypothesis. This data should be compared with the research output about SARS-CoV-2.

To our best knowledge, there is no report concerning medical scientists’ productivity related to COVID-19. We showed that authors from countries with higher HDI produced more articles than authors from less developed areas. Previous publications also confirmed this disturbing trend [14]. There is a risk that developing countries generate less new knowledge on COVID-19, which could eventually lead to their further scientific marginalization and poor description of the pandemic in these areas [15,16]. We also showed that in most cases the country ranking based on COVID-19 publication is similar to SCImago country rank. This suggests that authors coming from countries that normally produced the highest number of publications were also the most versatile in switching their scientific work related to the ongoing novel coronavirus pandemic. It may also show that research funders from these countries can quickly and efficiently provide scientists with money needed for specific research in emergency situations. There is also a possibility that the number of articles produced by authors from different countries varies not only because of lower HDI and severity of COVID-19 pandemics. We have to consider the possible impact of factors such as armed conflicts, clinical engagement of physicians, etc. Moreover, school closure may cause female researchers to leave scientific work and take care of their children [17].

Unsurprisingly, most of the articles were published in journals from the general category “Medicine,” and then “Infectious Diseases.” Further, the highest numbers of articles were published in categories “Cardiology and Cardiovascular Medicine” and “Dermatology.” In fact, COVID-19 may cause heart damage [18] and skin manifestations [19]. However, it may be suspected that more publications will be published in journals with categories such as “Public Health, Environmental and Occupational Health” and “Anesthesiology and Pain Medicine,” which will reflect the impact of the virus on public health and the emerging progress on intensive therapy on patients with severe COVID-19.

Strengths

First, entire MEDLINE was analyzed using an open API, and the economic and epidemiologic contexts were integrated in the calculations. The data we provided suggest that the level of development and scientific productivity prior to the ongoing pandemic determined the efficacy and rate of producing knowledge about a new, unknown danger. We may assume that future healthcare crises will also be better researched by countries with higher level of development, which are caring for their scientific productivity. This work generates further hypotheses. One of them is that greater spending on research proportionately associates with scientific productivity at the time of a public healthcare crisis [20]. We may also ask whether scientific productivity is associated with better results in fighting with the COVID-19 pandemic.

Limitations

The study has several limitations. First, we analyzed only articles accessible via PubMed, which include the MEDLINE database and papers included in the National Library of Medicine catalogue [21]. PubMed only recently started to include preprints in search results. Second, we did not weight the importance of the research by journals’ criteria, articles’ citations, or altmetrics. These features may additionally distinguish most essential papers. Finally, we analyzed a limited number of factors that may be associated with the article’s productivity.

## Conclusions

Most of early COVID-19 research output came from highly developed countries strongly affected by the pandemic. We believe that more researches on scientific productivity during the later months of the pandemics should be performed. It is also important to further investigate the factors that determine the number of publications coming from different countries. Our study, however, had a limitation. We did not present the information about scientific expenditure on COVID-19, which could be another interesting topic to cover in further research.

## Appendices

**Table 3:** Top 20 journal categories with the top 10 countries with highest number of articles on COVID-19 to 30 June 2020 on each category.

No	Medicine n = 5,038	Infectious Diseases n = 2,204	Cardiology and Cardiovascular Medicine n = 1,170	Dermatology n = 926	Public Health, Environmental and Occupational Health n = 497	Neurology n = 919	Gastroenterology n = 889	Anesthesiology and Pain Medicine n = 869	Biochemistry, Genetics, and Molecular Biology n = 1,008	Critical Care and Intensive Care Medicine n = 1,036
1	China n = 1,975	China n = 1,418	Italy n = 383	Italy n = 293	China n = 158	USA n = 244	Italy n = 243	USA n = 227	China n = 259	China n = 377
2	USA n = 835	Italy n = 388	China n = 273	Spain n = 118	Italy n = 66	Italy n = 187	China n = 169	China n = 154	USA n = 222	USA n = 150
3	Italy, UK n = 758	USA n = 362	USA n = 260	USA n = 110	USA n = 57	China n = 105	USA n = 159	UK n = 144	Italy n = 134	Italy n = 141
4		France n = 191	Spain n = 161	India n = 97	Iran n = 45	France n = 97	UK n = 118	Italy n = 117	France n = 109	France n = 137
5	India n = 522	Japan n = 155	UK n = 152	China n = 87	France n = 41	UK n = 72	France n = 69	Australia n = 45	UK n = 90	Germany n = 49
6	France n = 409	Iran n = 131	Germany n = 113	France n = 75	India n = 35	Spain n = 63	Germany, Spain n = 30	Germany n = 44	Germany n = 56	UK n = 38
7	Iran n = 314	UK n = 113	Australia n = 105	Turkey n = 59	UK n = 29	India n = 62		Singapore n = 38	Spain n = 45	Netherlands n = 30
8	Germany n = 218	Canada n = 97	India n = 98	Germany n = 34	Brazil n = 25	Germany n = 36	Japan n = 25	India n = 36	India n = 36	Canada n = 28
9	Spain n = 209	India n = 81	Canada n = 93	UK n = 27	Colombia n = 23	Canada n = 24	Australia, Belgium n = 23	Canada, Spain n = 32	Canada n = 33	Spain n = 23
10	Switzerland n = 180	Germany n = 79	France n = 90	Iran n = 26	Australia n = 18	Brazil n = 24			Japan n = 24	Belgium, India, Singapore n = 21
No	Internal Medicine n = 623	Hematology n = 932	Pediatrics, Perinatology, and Child Health n = 693	Surgery n = 497	Multidisciplinary n = 413	Immunology n = 806	Health Policy n = 428	Radiology, Nuclear Medicine, and Imaging n = 526	Epidemiology n = 375	Otorhinolaryngology n = 531
1	Italy, USA n = 131	Italy n = 214	China n = 182	Italy n = 184	China n = 179	China n = 220	USA n = 102	China n = 201	China n = 77	USA n = 265
2		USA n = 150	Italy n = 164	France n = 104	USA n = 71	Italy n = 170	China n = 93	Italy n = 101	France,Italy n = 53	France n = 110
3	India n = 89	UK n = 135	India n = 121	USA n = 74	Italy,UK n = 34	USA n = 132	UK n = 81	UK n = 66		Italy n = 58
4	China n = 71	France n = 114	USA n = 59	Brazil n = 35		Spain n = 84	Canada n = 37	Iran n = 51	UK n = 49	China, UK n = 22
5	Australia n = 48	China n = 101	UK n = 37	UK n = 22	Germany n = 29	France n = 51	Italy n = 35	USA n = 36	Germany n = 39	
6	UK n = 41	Spain n = 57	France, Spain n = 34	India n = 20	France n = 18	Germany n = 48	Australia n = 30	Japan n = 17	Netherlands n = 31	Brazil n = 18
7	Canada n = 34	Brazil, Germany, Netherlands n = 41		China, Germany n = 15	Japan n = 13	UK n = 34	India n = 17	Canada n = 15	Canada n = 25	India n = 12
8	Spain n = 24		Australia n = 30		Australia, Spain n = 12	Poland n = 28	France n = 12	Germany n = 14	USA n = 20	Germany n = 9
9	France n = 20		Germany n = 17	Spain, Turkey n = 14		Turkey n = 20	Iran n = 11	Spain n = 13	Australia n = 16	Spain n = 8
10	Brazil, Indonesia n = 17	Canada n = 38	Turkey n = 15		Switzerland n = 11	Australia n = 19	Belgium n = 10	France n = 12	Spain n = 12	Iran n = 7
